# Contractile Activity Regulates Inducible Nitric Oxide Synthase Expression and NO_i_ Production in Cardiomyocytes via a FAK-Dependent Signaling Pathway

**DOI:** 10.1155/2012/473410

**Published:** 2012-07-26

**Authors:** Miensheng Chu, Yevgeniya Koshman, Rekha Iyengar, Taehoon Kim, Brenda Russell, Allen M. Samarel

**Affiliations:** ^1^Cardiovascular Institute, Loyola University Chicago Stritch School of Medicine, 2160 South First Avenue, Maywood, IL 60153, USA; ^2^Department of Physiology and Biophysics, University of Illinois at Chicago, Chicago, IL 60612, USA

## Abstract

Intracellular nitric oxide (NO_i_) is a physiological regulator of excitation-contraction coupling, but is also involved in the development of cardiac dysfunction during hypertrophy and heart failure. To determine whether contractile activity regulates nitric oxide synthase (NOS) expression, spontaneously contracting, neonatal rat ventricular myocytes (NRVM) were treat with L-type calcium channel blockers (nifedipine and verapamil) or myosin II ATPase inhibitors (butanedione monoxime (BDM) and blebbistatin) to produce contractile arrest. Both types of inhibitors significantly reduced iNOS but not eNOS expression, and also reduced NO_i_ production. Inhibiting contractile activity also reduced focal adhesion kinase (FAK) and AKT phosphorylation. Contraction-induced iNOS expression required FAK and phosphatidylinositol 3-kinase (PI(3)K), as both PF573228 and LY294002 (10 **μ**M, 24 h) eliminated contraction-induced iNOS expression. Similarly, shRNAs specific for FAK (shFAK) caused FAK knockdown, reduced AKT phosphorylation at T308 and S473, and reduced iNOS expression. In contrast, shRNA-mediated knockdown of PYK2, the other member of the FAK-family of protein tyrosine kinases, had much less of an effect. Conversely, overexpression of a constitutively active form of FAK (CD2-FAK) or AKT (Myr-AKT) reversed the inhibitory effect of BDM on iNOS expression and NO_i_ production. Thus, contraction-induced iNOS expression and NO_i_ production in NRVM are mediated via a FAK-PI(3)K-AKT signaling pathway.

## 1. Introduction

Intracellular nitric oxide (NO_i_) is a free radical that is synthesized by a family of nitric oxide synthases (NOSs), consisting of endothelial NOS (eNOS or NOS1), inducible NOS (iNOS or NOS2), and neuronal NOS (nNOS or NOS3) isoforms. All three isoforms are expressed in cardiomyocytes. eNOS and nNOS are constitutively expressed, whereas iNOS is up-regulated under pathological conditions such as sepsis, hypertension, hypertrophy and heart failure [1, 2]. Thus, NO_i_ production plays an important role in regulating cardiomyocyte function during excitation-contraction coupling, mitochondrial respiration, hypertrophic remodeling, apoptosis, and myocardial regeneration.

The local effects of endogenous NO_i_ production on cardiomyocyte structure and function are dependent upon the localization of the different NOS isoforms. For instance, eNOS is associated with caveolin-3 within caveoli, where it locally regulates ß-adrenergic receptor stimulation [3]. nNOS is associated with sarcoplasmic reticulum (SR) membranes, where it modulates SR Ca^2+^  uptake and release [2–4]. During different pathological conditions, NO_i_ production shifts from spatially and temporally regulated NO_i_ production to dysregulated, excessive release. The relative abundance of NOS expression and activity in diseased hearts also changes, with nNOS and iNOS being upregulated, whereas eNOS is downregulated [5]. During these pathological states, iNOS is localized throughout the cytoplasm and increased iNOS expression and NO_i_ production then contribute to myocardial dysfunction and reduced myocardial responsiveness to ß-adrenergic stimulation [6–8]. Previous studies have demonstrated that iNOS expression is significantly increased in the intact, volume-overloaded heart [7], which is mediated in part by systemic production of TNF-*α*, IL-1*β*, angiotensin II, and other cytokines [1, 9]. However, iNOS expression and NO_i_ production can also be induced *ex vivo* by static stretch of cultured neonatal rat ventricular myocytes (NRVM) [10], suggesting the involvement of a mechanosensitive signaling pathway in cardiomyocytes that operates independently of circulating growth factors and cytokines. Indeed, cardiomyocytes can directly transduce physical forces into biochemical signals and generate appropriate responses leading to alterations in cellular structure and function [11]. Costameres (and focal adhesions, their functional equivalent in cultured cells) are important subcellular structures responsible for mechanotransduction in cardiomyocytes (for review, see [12]). A complex signaling web connects mechanosensory and growth factor-dependent signal transduction pathways in cardiomyocytes, and many different downstream effectors are activated in response to mechanical loading. Mechanotransduction via integrins and their accessory nonreceptor protein tyrosine kinases (focal adhesion kinase (FAK) and proline-rich tyrosine kinase 2 (PYK2)) resembles the downstream signals generated following activation of receptor tyrosine kinases, and their interconnecting signaling pathways share critical components with signaling pathways activated in response to peptide growth factors and cytokines [13]. These responses include acute alterations in contractile function, as well as long-term, structural changes in cardiomyocyte size, shape, and gene expression. 

In a previous study, we demonstrated that electrical pacing of quiescent, freshly isolated adult cat ventricular myocytes acutely increased NO_i_  production by a mechanochemical signaling pathway that required Ca^2+^-calmodulin (CaM), phosphatidylinositol-3-kinase (PI(3)K), and AKT [14]. Based upon relatively specific small-molecule inhibitors of the various NOS isoforms, acute stimulation of NO_i_ production by electrical pacing and mechanical activity involved both eNOS and nNOS. In this paper, we examined whether cardiomyocyte contractile activity also regulated NO_i_ production over a longer time period, via the contraction-dependent upregulation of iNOS expression in spontaneously contracting NRVM. We also investigated the roles of FAK, PYK2, PI(3)K, and AKT in this process.

## 2. Methods

### 2.1. Reagents

 PC-1 tissue culture medium was obtained from BioWhittaker (Walkersville, MD, USA). Dulbecco's Modified Eagle Medium (DMEM) and Medium 199 were obtained from Gibco BRL (Grand Island, NY, USA). FAK, iNOS, eNOS and C-terminal PYK2/CRNK monoclonal antibodies (mAb) were purchased from BD Transduction Laboratories (San Jose, CA, USA). Phospho-specific FAK-Y397 and FAK-Y577 polyclonal (pAb) antibodies were purchased from BioSource International, Camarillo, CA. Phospho-specific PYK2-Y402, AKT-T308_,_ AKT-S473, and total AKT pAb were purchased from Cell Signaling Technology (Danvers, MA, USA). N-terminal PYK2 pAb was obtained from BioLegend (San Diego, CA, USA). GAPDH mAb was obtained from Novus Biologicals, Littleton, CO, USA. Horseradish-peroxidase-conjugated goat anti-rabbit and goat anti-mouse IgGs were obtained from BioRad (Hercules, CA, USA). PF573228 and blebbistatin were obtained from Tocris Bioscience (Minneapolis, MN, USA). All other reagents were of the highest grade commercially available and were obtained from Sigma (St. Louis, MO, USA) and Baxter S/P (McGaw Park, IL).

### 2.2. Cell Culture

 Animals used in these experiments were handled in accordance with the Guiding Principles in the Care and Use of Animals, approved by the Council of the American Physiological Society. NRVMs were isolated from the hearts of 2-day old Sprague-Dawley rats by collagenase digestion, as previously described [15]. Cells were preplated for 1 h in serum-free PC-1 medium to reduce nonmyocyte contamination. The nonadherent NRVMs were then plated at a density of 1600 cells per mm^2^ onto collagen-coated 60 mm dishes and left undisturbed in a 5% CO_2_ incubator at 37°C for 36 h. Unattached cells were removed by aspiration, washed twice in HBSS, and the attached cells were maintained in a solution of DMEM/Medium 199 (4 : 1) containing antibiotic/antimycotic solution. At this density, spontaneous contractile activity (~100–150 beats per min) was visible within 24 h of plating. NRVMs were then infected (24 h) with replication-defective adenoviruses (Adv) diluted in DMEM/Medium 199. Medium was replaced with virus-free DMEM/Medium 199, treated with inhibitors or cultured for an additional 6–72 h.

### 2.3. Adenoviral Constructs

 Advs expressing shRNAs specific for rat FAK (shFAK), rat PYK2 (shPYK2), and firefly luciferase (shLuc) were generated as previously described [16]. To inhibit FAK-dependent signal transduction, NRVMs were infected (5 moi, 24 h) with replication-defective adenoviruses (Adv) expressing either GFP-FRNK [17] or Y397F-FAK [18], the latter of which was kindly provided by Dr. T. Kasahara, Kyoritsu College of Pharmacy, Tokyo, Japan. To inhibit PYK2-dependent signaling, a replication-defective Adv-expressing FLAG-tagged, human Cell Adhesion Kinase-*β* Related Non-Kinase (CRNK) was kindly provided by Dr. Andrey Sorokin, Medical College of Wisconsin [19]. FAK-dependent signaling was increased using replication-defective Adv expressing either wildtype (WT) FAK [20] or a “constitutively active” FAK fusion protein (CD2-FAK) [21] which was constructed as previously described [16]. AKT-dependent signaling was increased using a replication-defective Adv-expressing either WT-AKT, or constitutively active AKT (Myr-AKT), which were kindly provided by Dr. K. Walsh, Tufts University School of Medicine, Boston, MA, USA [22]. In each experiment, a replication-defective Adv expressing either GFP [17] or nuclear-encoded *β*-galactosidase (neßgal) [23] was used to control for nonspecific effects of Adv infection. All Advs were propagated in HEK293 cells and purified by CsCl gradient centrifugation. The multiplicity of viral infection (moi) was determined by viral dilution assay in HEK293 cells grown in 96 well clusters. At moi of 5–10, >95% of the cells were infected, as determined by Adv-neßgal infection and X-gal staining, and there were no cytotoxic effects of Adv infection during the 24–72 h following Adv infection.

### 2.4. SDS-PAGE and Western Blotting

 NRVMs were homogenized in lysis buffer [24], and equal amounts of extracted proteins (50–100 *μ*g) were separated by SDS-PAGE and Western blotting on 10% polyacrylamide gels. Primary antibody binding was detected with horseradish peroxidase-conjugated goat anti-mouse or goat anti-rabbit secondary antibodies and visualized by enhanced chemiluminescence (Pierce Biotechnology, Rockford, IL, USA). Developed films were then scanned on a HP Deskjet 4890 Scanner, and band intensity was quantified using UN-SCAN-IT Gel Software, Ver. 6.1 (Silk Scientific, Orem, UT). In all experiments, band intensity obtained with the phosphospecific antibody was divided by the band intensity for total protein (phosphorylated + nonphosphorylated) and then normalized to the intensity of the control sample.

### 2.5. NO_i_ Measurements

 Measurements of NO_i_ production were obtained using the fluorescent NO-sensitive dye 4,5-diaminofluorescein diacetate (DAF-2 DA) [25, 26] as previously described [27]. Cells were incubated with membrane-permeant DAF-2 DA (2 *μ*M; 40 min) at room temperature in 3 mL standard Tyrode solution containing 100 *μ*M L-arginine. Cells were subsequently washed for 30 min. DAF-2 fluorescence was excited at 480 nm and emitted cellular fluorescence was recorded at 540 nm. Cellular DAF-2 fluorescence intensity (F) was quantified with Image J Software. Control measurements of NO_i_ obtained in different experiments were grouped together and compared to drug treatment or adenoviral infection groups.

### 2.6. Data Analysis

 Results were expressed as means ± SEM. Normality was assessed using the Kolmogorov-Smirnov test. Data were compared using one-way ANOVA followed by Student-Newman-Keuls test, one-way ANOVA on Ranks followed by Dunn's test, Student-Newman-Keuls test, or paired t-test, where appropriate. Differences among means were considered significant at *P* < 0.05. Data were analyzed using SigmaPlot for Windows, Ver. 9.0 (Systat Software, San Jose, CA, USA).

## 3. Results

### 3.1. Contractile Activity is Essential for iNOS Expression in NRVM

**Figure 1:**
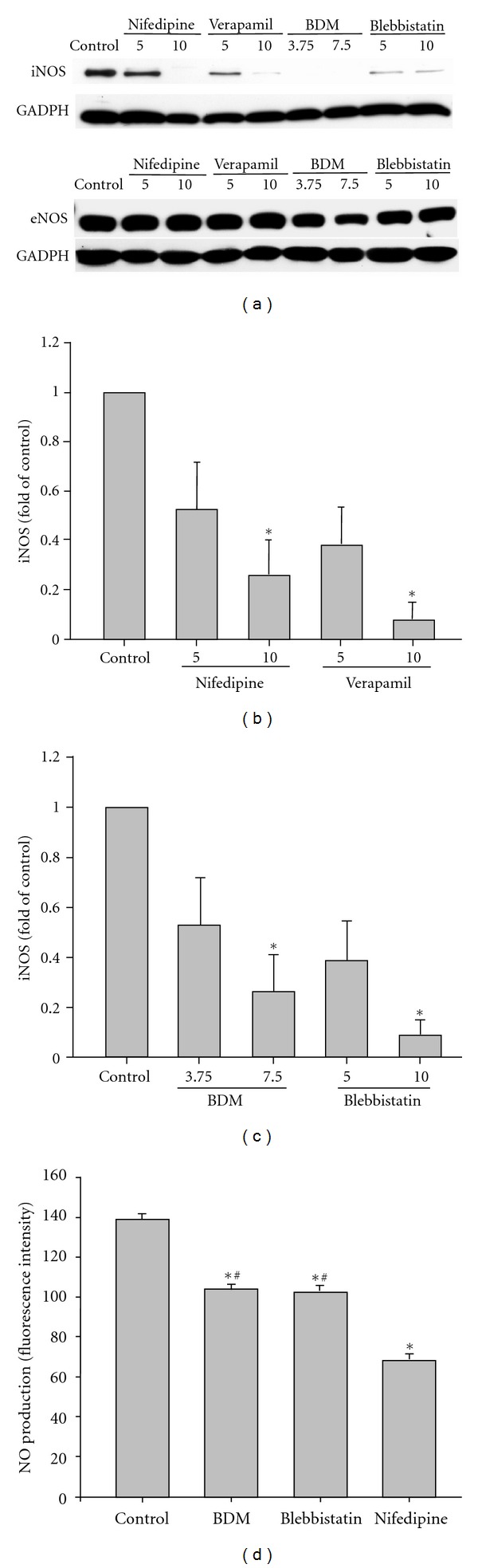
Contractile activity is essential for iNOS expression. (a) NRVMs were treated with L-type calcium channel blockers (nifedipine 5–10 *μ*M or verapamil 5–10 *μ*M) or myosin II ATPase inhibitors (butanedione monoxime (BDM) 3.75–7.5 mM or blebbistatin 5–10 *μ*M) for 24 h. Cell extracts (50 *μ*g total protein per lane) were then separated by SDS-PAGE and Western blotting with antibodies specific for iNOS, eNOS, and GAPDH. (b and c) The quantitative results of 5 experiments are depicted. *P < 0.05 versus. untreated, control cells. (d) NO_i_ was measured under control conditions and in the presence of 7.5 mM BDM, 10 *μ*M blebbistatin or 10 *μ*M nifedipine. The quantitative results of 5 experiments are depicted. *P < 0.05 versus untreated, control cells. ^#^ *P* < 0.05 versus Nifedipine-treated cells.

Spontaneously contracting NRVMs were treated with the L-type calcium channel blockers nifedipine and verapamil (which inhibit both spontaneous [Ca^2+^]_i_ transients and contractile activity) or the myosin II ATPase inhibitors butanedione monoxime (BDM) and blebbistatin (which block contractile activity but have relatively little effects on spontaneous [Ca^2+^]_i_ transients) [14, 28–30]. iNOS and eNOS expression was then examined by SDS-PAGE and Western blotting under basal conditions and in response to contractile arrest. As seen in Figure 1(a), NRVM expressed substantial quantities of both eNOS and iNOS under basal conditions. Treatment with the various inhibitors of spontaneous contractile activity all significantly reduced iNOS expression in a dose-dependent fashion (Figures 1(b) and 1(c)), with little or no effect on eNOS expression levels (Figure 1(a)). As both types of contractile inhibitors substantially reduced iNOS expression, these results suggested that mechanical activity per se, rather than a Ca^2+^-dependent, contraction-independent signaling pathway was required. Next, we measured NO_i_ production using DAF-2-DA. As shown in Figure 1(d), nifedipine, BDM and blebbistatin all reduced NO_i_ production as compared to spontaneously contracting, control NRVM. Interestingly, nifedipine reduced NO_i_ production to a greater extent than either BDM or blebbistatin, suggesting that at the time of NOi measurement, both [Ca^2+^]_i_ and mechanical activity contributed to enhanced NO_i_ production. The partial [Ca^2+^]_i_ dependence of NO_i_ production is consistent with the known role of Ca^2+^-CaM in regulating eNOS activity [31].

### 3.2. Contraction-Dependent iNOS Expression Requires FAK and PI(3)K

**Figure 2:**
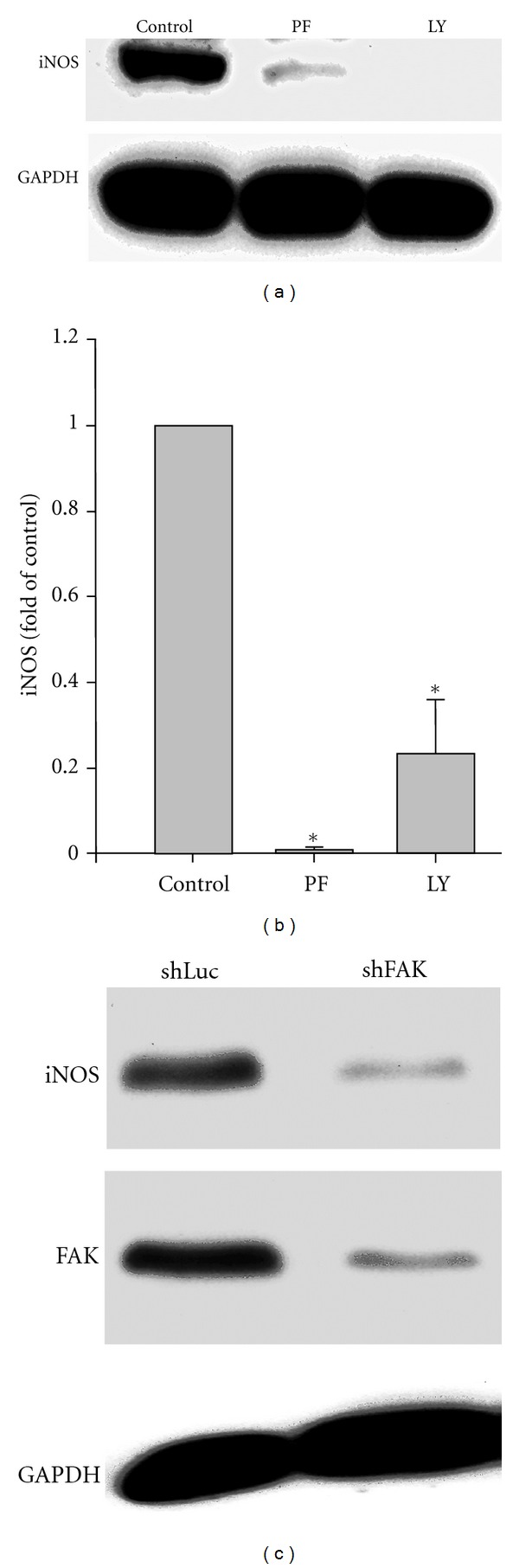
Contraction-induced iNOS expression is regulated by FAK and AKT. (a) NRVMs were maintained in control medium (con) or treated with PF 573228 (PF, 10 *μ*M) or LY 294002 (LY, 10 *μ*M) for 24 h. Cell extracts (50 *μ*g total protein per lane) were then separated by SDS-PAGE and Western blotting with antibodies specific for iNOS and GAPDH. (b) The quantitative results of 5 experiments are depicted. *P < 0.05 versus control. (c) NRVM were infected, (20 moi) with Adv-expressing shRNAs for luciferase (shLuc) or FAK (shFAK) and then maintained under control conditions for 72 h. Equal amounts of cell extracts (50 *μ*g total protein per lane) were then separated by SDS-PAGE and Western blotting with antibodies specific for FAK, iNOS, and GAPDH.

To investigate the role of FAK and PI(3)K in regulating contraction-dependent iNOS expression, NRVMs were first treated with PF573228 or LY294002, which are potent and highly selective inhibitors of FAK [32] and PI(3)K [33] activities, respectively. As seen in Figures 2(a) and 2(b), both agents substantially reduced iNOS levels in NRVM. Of note, at the concentrations used here, neither agent appeared to affect spontaneous contractile activity in these high-density cultures. To confirm the role of FAK in regulating iNOS expression, NRVMs were also infected with Adv-expressing shRNAs specific for FAK (shFAK) [16]. An Adv-expressing a shRNA specific for firefly luciferase (shLuc) was used to control for nonspecific effects of Adv infection and shRNA expression. As seen in Figure 2(c), shFAK but not shLuc substantially reduced FAK expression and also reduced iNOS expression. iNOS expression was also reduced by using 2 other inhibitors of FAK-dependent signaling (i.e., GFP-FRNK and Y397F-FAK; data not shown).

**Figure 3:**
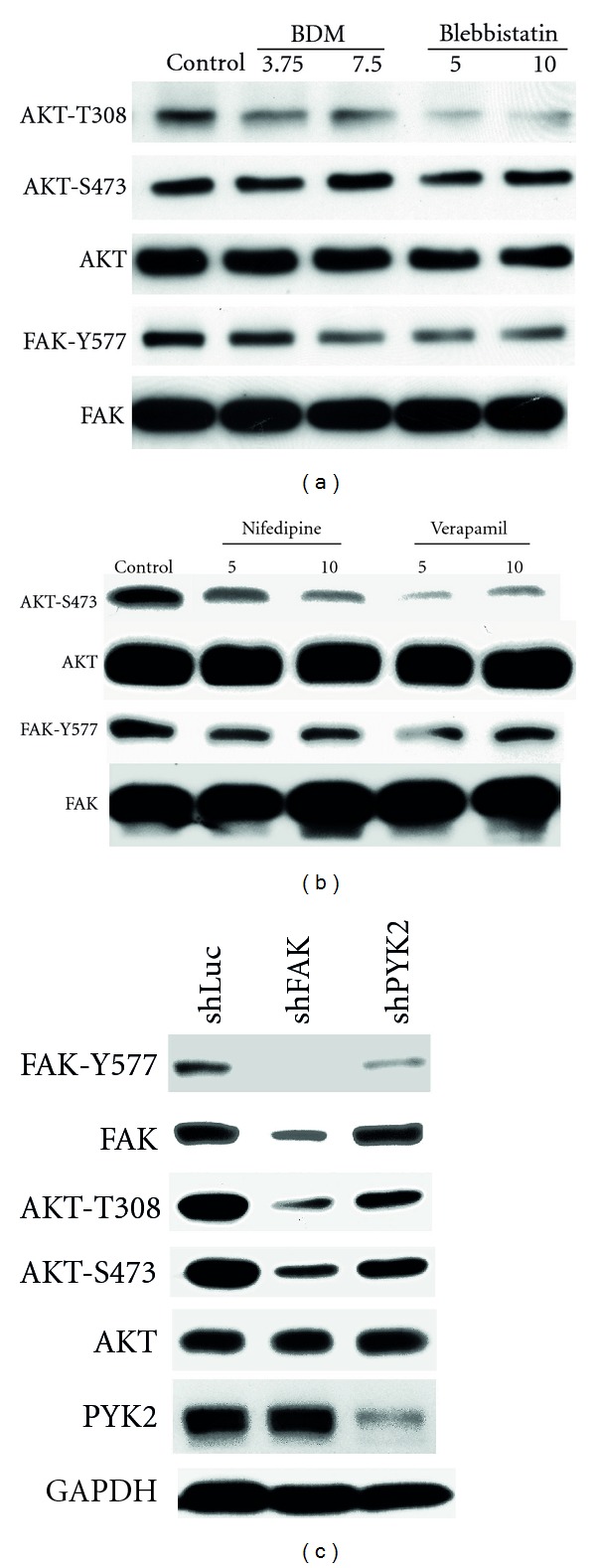
FAK regulates contraction-dependent activation of AKT. (a) NRVMs were treated with myosin II ATPase inhibitors (BDM or blebbistatin (Bleb)) or (b) L-type calcium channel blockers (nifedipine or verapamil) for 24 h. Cell extracts (50 *μ*g total protein per lane) were then separated by SDS-PAGE and Western blotting with antibodies specific for FAK phosphorylated at Y577, total FAK, AKT phosphorylated at T308 and S473, and total AKT. (c) NRVMs were infected, (20 moi) with Adv-expressing shRNAs for luciferase (shLuc), FAK (shFAK), or PYK2 (shPYK2) and then maintained under control conditions for 72 h. Equal amounts of cell extracts (50 *μ*g total protein per lane) were then separated by SDS-PAGE and Western blotting with antibodies specific for FAK phosphorylated at Y397, total FAK, AKT phosphorylated at T308 and S473, total AKT, total PYK2, and GAPDH.

**Figure 4:**
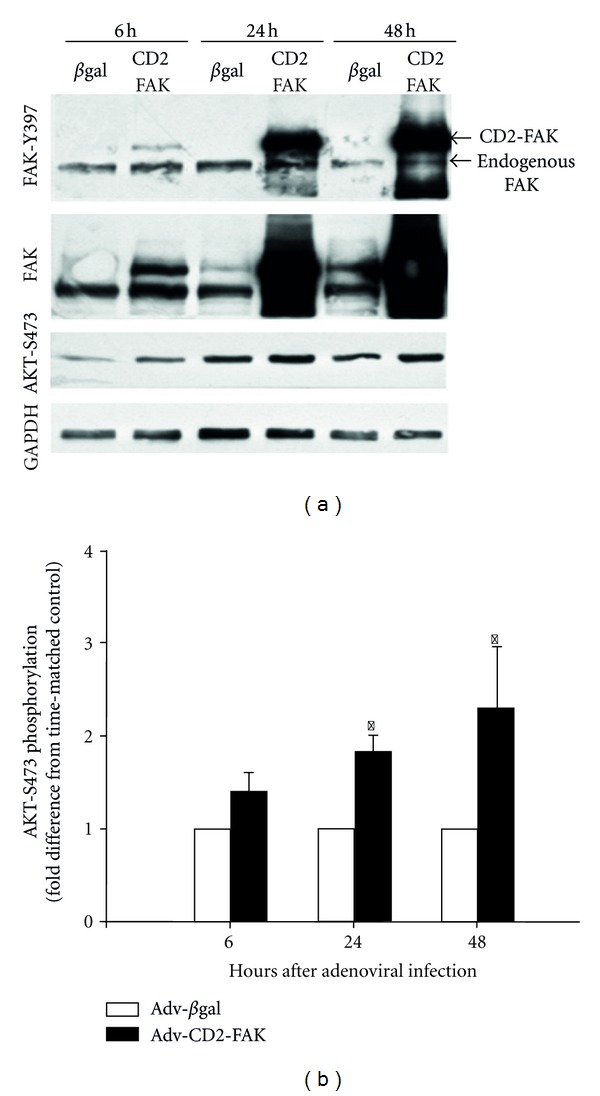
AKT activation is mediated by FAK. (a) NRVMs were infected (5 moi, 6–48 h) with Adv-expressing ne*β*gal or CD2-FAK. Cell extracts (50 *μ*g total protein per lane) were then separated by SDS-PAGE and Western blotting with antibodies specific for FAK phosphorylated at Y397, total FAK, AKT phosphorylated at S473, and total AKT. (b) The quantitative results of 9 experiments are depicted. *P < 0.05 versus time-matched, Adv-ne*β*gal cells.

Previous studies have demonstrated that FAK autophosphorylation at Y397 provides a docking site for the binding and tyrosine phosphorylation of the p85 subunit of PI(3)K, which is required for its activation in NRVM [34, 35] and other cell types [36–41]. PI(3)K activation in turn leads to the activation of AKT. As both FAK and PI(3)K activities appeared necessary to regulate contraction-dependent iNOS expression, we next examined whether FAK was also involved in regulating AKT. As seen in Figures 3(a) and 3(b), spontaneously contracting NRVM demonstrated high levels of basal AKT activation, as detected by Western blotting with antibodies specific for AKT phosphorylated at T308 and S473. In contrast, NRVMs arrested with L-type Ca^2+^ channel blockers or inhibitors of myosin ATPase demonstrated reduced FAK and AKT phosphorylation. shRNA-induced FAK knockdown also reduced AKT phosphorylation at both sites (Figure 3(c)). In contrast, shRNA-mediated knockdown of PYK2, the other member of the FAK family of protein tyrosine kinases, had much less of an effect on contraction-dependent AKT phosphorylation. Similar negative results were observed by overexpressing CRNK [42], the C-terminal inhibitor of PYK2 (data not shown). Finally, Adv-mediated overexpression of a “constitutively active” form of FAK (CD2-FAK) [21] was sufficient to further increase AKT phosphorylation in spontaneously contracting NRVMs (Figures 4(a) and 4(b)).

### 3.3. Rescue of iNOS Expression in Contractile-Arrested NRVMs

**Figure 5:**
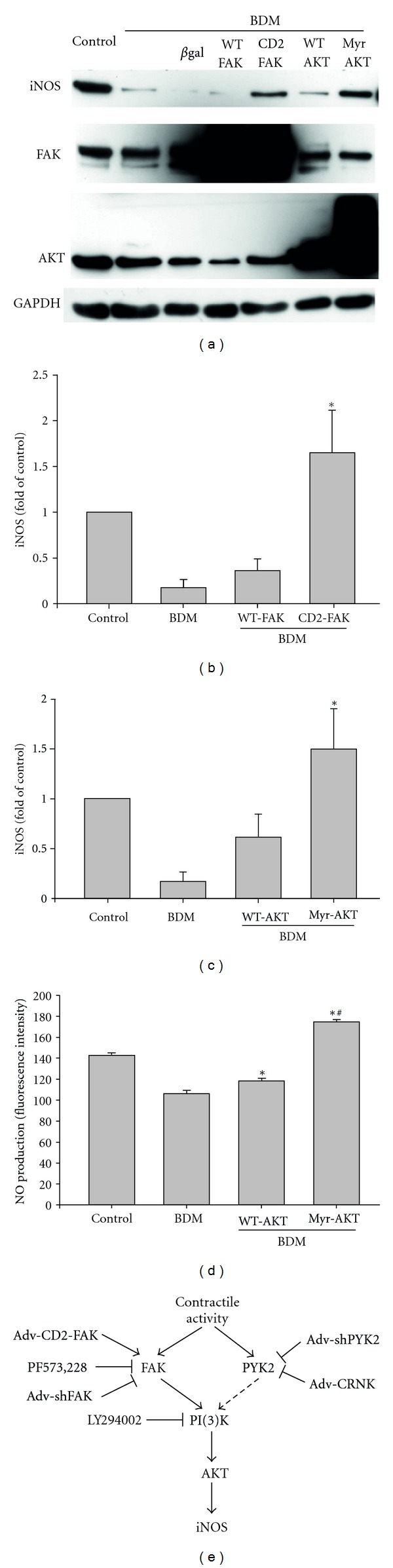
Rescue of BDM inhibition of iNOS expression by CD2-FAK and Myr-AKT. (a) NRVMs were pretreated with BDM (7.5 mM) for 24 h. To “rescue” the BDM inhibition of iNOS expression, some cells were then infected (48 h, 10 moi) with Adv-expressing ne*β*gal, WT-FAK, CD2-FAK, WT-AKT, or Myr-AKT with continued exposure to BDM. Cell extracts (50 *μ*g total protein per lane) were then separated by SDS-PAGE and Western blotting with antibodies specific for FAK, AKT, iNOS, and GAPDH. ((a) and (c)) The quantitative results of 5 experiments are depicted. *P < 0.05 versus WT-FAK or WT-AKT cells. (d) NRVMs were treated with 7.5 mM BDM for 24 h. To “rescue” the BDM inhibition of NO_i_ production, some cells were then infected (10 moi) with WT-AKT or Myr-AKT for 48 h with continued exposure to BDM. NO_i_ was then measured using DAF-2 DA. The quantitative results of 5 experiments are depicted. *P < 0.05 versus BDM, ^#^ *P* < 0.05 versus WT-AKT. (e) A multicomponent signaling pathway that links mechanical load to AKT activation and downstream signaling to iNOS is depicted.

 To further examine the role of FAK, PI(3)K, and AKT in regulating contraction-dependent iNOS expression, we attempted to “rescue” BDM inhibition of iNOS expression by overexpressing wildtype (WT) FAK, “constitutively active” FAK (i.e., CD2-FAK), WT-AKT, or a constitutively active form of AKT (Myr-AKT) [22]. As seen in Figures 5(a) and 5(b), overexpression of WT-FAK had no effect on the BDM-mediated inhibition of iNOS expression. However, CD2-FAK overexpression rescued iNOS expression following BDM treatment. Of note, BDM treatment alone reduced iNOS expression to ~20% of spontaneously contracting NRVM. Overexpression of CD2-FAK in BDM-arrested NRVM caused a relative ~8-fold increase in iNOS expression, thus restoring its expression to levels above that observed in contracting cells (Figures 5(a) and 5(b)). Similarly, Myr-AKT overexpression also reversed the inhibitory effect of BDM (Figures 5(a) and 5(c)). To confirm these results, we performed the rescue experiment on BDM-treated cells but measured NO_i_ production using DAF-2-DA. Both WT-AKT and Myr-AKT were sufficient to restore NO_i_ production under BDM inhibition (Myr-AKT > WT-AKT) (Figure 5(d)). These results confirmed that FAK and AKT are both necessary and sufficient for contraction-mediated iNOS expression and NO_i_ production in NRVM.

## 4. Discussion

As demonstrated in this report and our previous study [14], cardiomyocyte contractile activity is an important factor that regulates NOSs and NO_i_ production via Ca^2+^-dependent and Ca^2+^-independent signaling pathways. As demonstrated in Figure 1, nifedipine reduced NO_i_ production to a greater extent than either BDM or blebbistatin. Previous studies have also shown that acute mechanical stimulation, such as, by sheer stress [43, 44] and static stretch [45] stimulate eNOS-dependent NO_i_ production in endothelial cells. A similar effect of acute mechanical loading on NO_i_ production was observed in cardiomyocytes [14, 31, 46, 47]. However, in these studies, the acute load-dependent phosphorylation of eNOS at S1177 was likely the responsible mechanism. Furthermore, eNOS activity can be regulated via activation of PI(3)K-AKT or Ca^2+^-CaM signaling in different cell types [14, 44, 48]. In our study, the additive effect of inhibition of Ca^2+^ influx and contractile arrest led to a further reduction in NO_i_ production as compared to the inhibition of mechanical activity alone. These results are consistent with other observations that Ca^2+^-dependent and Ca^2+^-independent NOSs are both involved in mechanical stimulation-induced NO_i_ production. In our previous study, we showed that either decreased (BDM or blebbistatin) or increased (EMD 57033 or CGP 48506) contractile strength affected changes in field stimulation-induced NO_i_ production without changing Ca^2+^ influx (12). Here we demonstrate that contractile activity can also induce iNOS gene expression and NO_i_ production through a FAK-PI(3)K-AKT signaling pathway without interfering with Ca^2+^ handling. A schematic diagram outlining this signaling pathway is depicted in Figure 5(e).

Previous studies have shown that mechanical stretch can induce both iNOS and eNOS expression in NRVM [10, 49]. However in the present study, we could only detect a significant reduction in iNOS (but not eNOS) expression when cells were treated with L-type Ca^2+^ channel blockers or myosin II ATPase inhibitors. These results suggest that load-induced eNOS upregulation may be mediated by a different signaling mechanism. Liao et al. [10] also indicated that the stretch-induced Ca^2+^ signal was very likely the trigger for subsequent NO_i_ signaling in cardiomyocytes, and the NO_i_ signal further amplified itself by increasing iNOS expression. However, both types of inhibitors could not completely eliminate iNOS expression or NO_i_ production, suggesting that contraction-induced iNOS expression may also be subjected to NO_i_ autoregulation. 

Our experiments utilized NRVM plated at high density (~1 × 10^5^ cells/cm^2^) in order to enhance spontaneous contractile activity and increase iNOS expression. Under these conditions, FAK and AKT were also highly phosphorylated, but their phosphorylation could be reduced by inhibitors of contractile activity. Hines et al. [50] have also shown that dense myocyte cultures displayed higher metabolic activity and contraction rates as compared to cells plated at low density. Contractility may also regulate FAK, PI(3)K, and AKT activity in vivo. For instance, obese type 2 diabetic (db/db) mice have significantly reduced cardiac contractility and depressed PI(3)K-AKT signaling [51]. Conversely, acute pressure overload increased FAK, PI(3)K, and AKT activation in the intact heart [52]. These data suggest that cardiomyocyte contractility can activate several downstream signaling cascades in order to maintain cardiac function. 

Although FAK appears both necessary and sufficient to induce AKT activation in NRVM, the intermediary steps linking contractile activity to FAK and AKT activation remain unclear. As indicated in our previous paper, contraction-induced NO_i_ production required PI(3)K and AKT, as the highly specific PI(3)K inhibitor LY294002, or overexpression of a dominant-negative mutant of AKT, partially blocked contraction-dependent NO_i_ production [14]. Similar results were obtained by Del Re et al. [35], who showed that static stretch-induced AKT activation required FAK and PI(3)K. Furthermore, we recently showed that adhesion-dependent AKT activation in adult atrial myocytes was also dependent on FAK and PI(3)K [53]. In this regard, there are several potential mechanisms whereby PI(3)K may be involved. First, previous studies in nonmuscle cells have indicated that the p85 regulatory subunit of PI(3)K is tyrosine phosphorylated by FAK and can directly bind to the FAK-Y397 autophosphorylation site, thereby activating the lipid kinase [36–41]. Wei and Vander Heide [34] showed that FAK and p85 formed a complex in unstimulated NRVMs, and their association increased in response to heat stress. The heat stress-induced complexation of FAK and p85 was associated with an increase in AKT phosphorylation, which was prevented by FRNK overexpression. Del Re et al. [35] also demonstrated a similar static stretch-induced complexation of FAK and p85 in NRVM. Thus, a direct interaction between FAK and PI(3)K appears to be operative in cardiomyocytes.

Alternatively, Taniyama et al. [54] have shown that angiotensin II stimulated the tyrosine phosphorylation and activation of PDK1 in vascular smooth muscle cells via a PYK2- and c-Src-dependent pathway. Guo et al. [55] have shown that in NRVM, a similar pathway is activated by H_2_O_2_, leading to downstream phosphorylation of AKT at both T308 and S473. Although we demonstrate that PYK2 is not necessary for contraction-induced AKT activation, it is conceivable that FAK, or the FAK/Src complex may function in a similar manner, thereby causing the tyrosine phosphorylation of PDK1 and subsequent AKT activation. Thus, the requirement for PI(3)K to mediate contraction-induced AKT activation would be conferred through the generation of phosphoinositides necessary for PDK1 and AKT recruitment to the cell membrane and not directly via formation of a FAK/PI(3)K complex. Of note, Xia et al. [41] demonstrated that ligation of *β*_1_ integrins (accomplished with an anti-*β*_1_ integrin antibody) activated both FAK and AKT in a Src- and PI(3)K-dependent manner, although FAK-p85 complexation could not be demonstrated. 

Several studies have used genetically modified animal models to address the function of iNOS in the heart. Three different research groups have shown that iNOS deletion improved ventricular function, reduced myocardial nitrotyrosine content, decreased apoptosis, and improved survival after experimental myocardial infarction [56–58]. Zhang et al. [59] also demonstrated that iNOS deletion protected against pressure overload-induced ventricular hypertrophy and heart failure, in association with decreased myocardial nitrotyrosine and 4-hydroxy-2-nonenal. Furthermore, Mungrue et al. [60] have shown that cardiac-specific iNOS overexpression caused increased myocardial peroxynitrite production, myocardial fibrosis, ventricular hypertrophy, heart failure, and sudden cardiac death. These studies suggest that iNOS has significant detrimental effects on cardiac function. However, other studies have shown contradictory results, in that iNOS deficiency did not reduce myocardial infarct-induced ventricular dysfunction or mortality [61]. In fact, other studies demonstrated that iNOS plays a cardioprotective role during myocardial ischemia/reperfusion [62, 63]. Thus, the function of iNOS in the cardiomyocyte remains unclear, and additional studies are needed to evaluate the role of contractile activity-induced iNOS expression in regulating cardiomyocyte function and cell survival.
